# Evolution of alpha-Gal synthesis - a growing risk for alpha Gal syndrome

**DOI:** 10.1080/07853890.2026.2728764

**Published:** 2026-09-03

**Authors:** Rita Vaz-Rodrigues, José de la Fuente

**Affiliations:** ^a^ Health and Biotechnology-SaBio, Instituto de Investigación en Recursos Cinegéticos IREC-CSIC-UCLM-JCCM, Ciudad Real, Spain; ^b^ Department of Veterinary Pathobiology, College of Veterinary Medicine, Oklahoma State University, Stillwater, Oklahoma, USA

**Keywords:** Alpha-Gal, allergy, arthropod, evolution, galactosyltransferase

## Abstract

**Background:**

Alpha-Gal syndrome (AGS) is a growing burden worldwide associated with arthropods and allergies with pathologies such as urticaria, digestive and anaphylactic reactions. These reactions commonly appear 2–6 h after consumption of mammalian meat and derived pharmaceuticals mediated by immunoglobulin E against the oligosaccharide alpha-Gal epitope.

**Objective:**

Despite the increasing incidence of AGS, it is underdiagnosed and requires information and initiatives to reduce disease risks and incidence while improving prevention and treatment.

**Content:**

Our hypothesis is that Galactosyltransferase associated with alpha-Gal synthesis appeared and evolved in bacteria during the Mesoproterozoic Era ca. 1000 million years ago (Mya). Then, in Mollusca during the Cambrian and in Arthropoda and Mammalia during the Triassic period associated with improved fitness in organisms repopulating the Earth after Permian-Triassic Great Dying extinction event but affected Hominidae leading to catastrophic selection in the Oligocene.

**Conclusions:**

With global rapidly changing climate and population dynamics, it should be considered that multiple arthropod species evolved to synthesize alpha-Gal as a guidance to develop preventive and control measures to reduce the risks of exposure to AGS. Accordingly, research should focus on the role of other organisms in addition to ticks in the evaluation and prevention of risks associated with the AGS with education for patients and medical staff to improve prevention and management of patients with AGS.

**Key messages**
- The alpha-Gal synthesis evolved across multiple Arthropoda, Mollusca and Mammalia
- Hominids developed to produce anti-alpha-Gal antibodies with protective capacity
- Alpha-Gal syndrome (AGS) in humans is associated with arthropods and allergic reactions
- Other organisms are potential overlooked sources of alpha-gal

## Introduction

Alpha-Gal syndrome (AGS) is mediated by immunoglobulin E (IgE) against the oligosaccharide galactose-alpha-1,3-galactose (alpha-Gal epitope, Galα1-3Galβ1–4GlcNAc-R) [1–4]. The AGS is a growing burden worldwide associated with arthropods and allergic reactions with pathologies such as urticaria, digestive disorders and anaphylaxis that commonly occur 2 to 6 h after consumption of alpha-Gal-containing mammalian meat and pharmaceuticals [1–4]. Despite the increasing incidence of AGS, it is underdiagnosed and requires information and initiatives to reduce disease risks and incidence while improving prevention and treatment [5].

The galactosyltransferases and alpha-galactosidase produce alpha-Gal modifications in proteins and lipids [1,2]. These modifications are associated with tick fitness, feeding and reproduction [1,2], and play a role in mammals except catarrhine primates (Old World monkeys, apes and humans) on tissue structural stability, protein folding and intracellular transport [3].

Although the AGS is mainly associated with ticks, cross-reactivity between wasp venom and tick proteins has been described and associated with sensitization in patients with AGS [6]. With preliminary evidence, it has been proposed that mosquitoes and tsetse flies may be associated with the AGS not only through alpha-Gal synthesis but also through IgE sensitization to allergens and alpha-Gal [7–9]. Furthermore, an inverse relationship has been reported between cases with AGS and cases with anaphylaxis to imported fire ants, *Solenopsis invicta*, associated with fire ants as predators of the lone star tick so they are geographically competing for territory and reducing tick and thus AGS prevalence [10].

Our hypothesis addressed in this article is how the alpha-Gal synthesis evolved with possible implications in the AGS. The hypothesis is that alpha-Gal synthesis appeared in bacteria during the Mesoproterozoic Era ca. 1000 millions years ago (Mya) and then evolved in Mollusca during the Cambrian and in Arthropoda and Mammalia during the Triassic period associated with improved fitness in new organisms repopulating the Earth after Permian-Triassic “Great Dying” extinction event and continued after the transition following the end-Cretaceous extinction with evolutionary implications.

## Discussion

**Figure 1.**
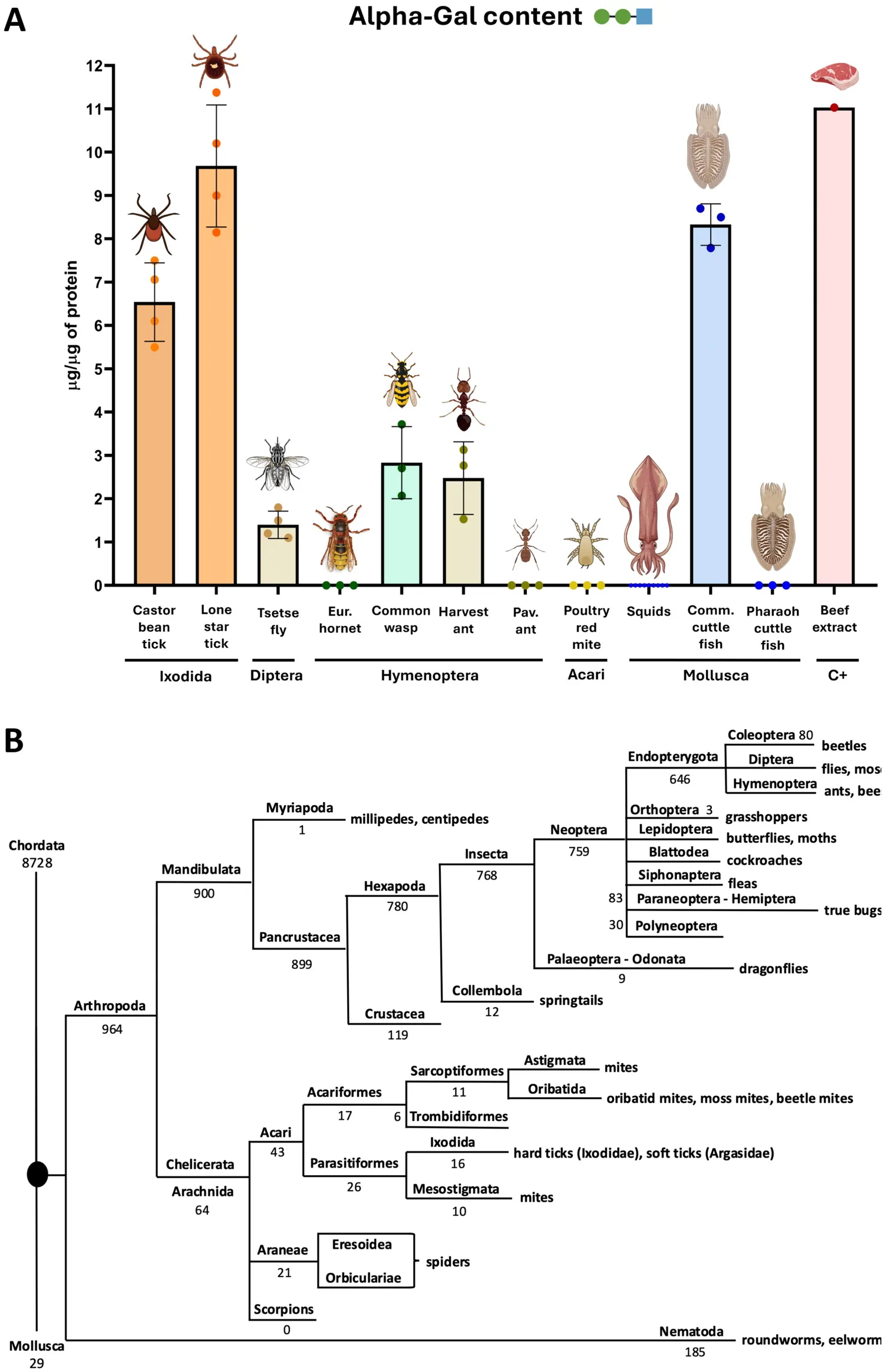
Alpha-Gal content in different organisms. (A) Alpha-Gal levels determined by in-house indirect ELISA assay as described previously [10]. The analysis was conducted in protein extracts from different taxa: Ixodida, Diptera, Hymenoptera, Acari and Mollusca. The analysis included samples from ticks *Ixodes ricinus* castor bean rick, *Amblyomma americanum* lone star tick, dipteran *Glossina fuscipes fuscipes* tsetse fly, subclass Acari poultry red mite *Dermanyssus gallinae*, order Hymenoptera *Tetramorium* sp. pavement ant, *Messor* sp. harvest ant, *Vespa crabro* European hornet, *Vespula vulgaris* common wasp, Cephalopod squids *Loligo reynaudii* Cape Hope squid, *Dosidicus gigas* Humboldt squid, *Illex coindetii* southern shortfin squid, *Sepia officinalis* common cuttlefish and *Sepia pharaonis* pharaoh cuttlefish. Beef extract was used as positive control (C+). (B) Phylogenetic analysis of alpha-gal synthase (Arthropoda and Nematoda). Data for Glycoprotein-N-acetylgalactosamine 3-beta-galactosyltransferase 1 (1,3-galactosyltransferase, GGTA1) was obtained in the UniProt database (https://www.uniprot.org/uniprotkb?query=α-1%2C3-galactosyltransferase ± Taxa; search on September 7, 2025). The number of sequences is shown for each cluster.

The synthesis of alpha-Gal has been mainly reported in Arthropoda including Diptera (mosquitoes, tsetse fly), Acari (chiggers), Hymenoptera (wasp, ant), Nematoda and Ixodida ticks [11–15] (Figure 1(A)). Mollusca Cephalopoda cuttlefish species are also positive for alpha-Gal (Figure 1(A)), but Scorpions, Orthoptera, Coleoptera and other Acari (poultry red mite), Hymenoptera (pavement ant, European hornet) and Mollusca (squids, pharaoh cuttle fish) are negative for alpha-Gal [12] (Figure 1(A)). The UniProt analysis of 1,3-galactosyltransferase, GGTA1 conducted in Arthropoda and Nematoda supports the presence of proteins in all taxa except for Scorpions, which have been reported as alpha-Gal negative [12] (Figure 1(B)). However, although Orthoptera and Coleoptera have been also reported negative for alpha-Gal [12], three and 80 proteins were registered in the UniProt database, respectively (Figure 1(B)). Therefore, it is possible that some organisms with potential galactosyltransferase activity do not contain this carbohydrate epitope on their tissues or saliva because among other factors, the enzymes involved in the final N-glycan maturation in the Golgi apparatus which are different between organisms are not active to complete the multi-step process [16].

**Figure 2.**
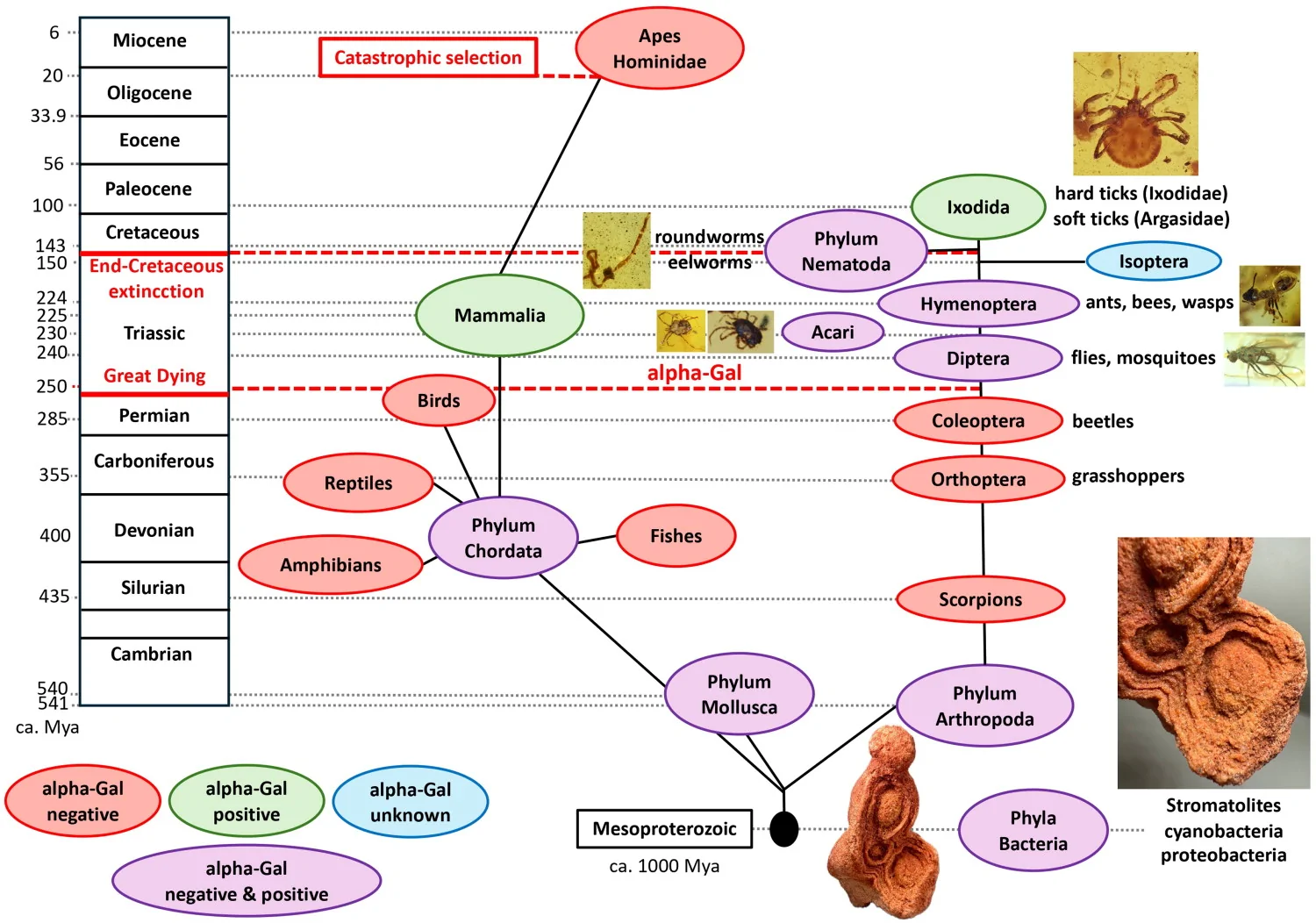
Evolution of alpha-Gal synthesis. Approximate origin (ca. Mya) for each cluster is shown. Data on alpha-Gal synthesis was collected from publications or generated in this study. Images of fossil inclusions shown to represent species with positive alpha-Gal synthesis are from J. de la Fuente author′s KGJ Collection (Ciudad Real, Spain).

Based on the oldest fossils on Earth, stromatolites (e.g. Mesoproterozoic period, ca. 1100 Mya, Sahara Desert, Morocco; Figure 2), composed primarily of cyanobacteria, provide information on the origin of bacteria and complex microbial communities [17,18]. They were the only major source of atmospheric oxygen critical for the development and evolution of more complex life. Other organisms such as viruses and microheterotrophs have been considered that had a role in lithification of modern microbial mats [17,19]. Microheterotrophs are small heterotrophic microorganisms primarily composed of bacteria, flagellates and ciliates that obtain energy by consuming organic matter with a key role in decomposition and nutrient cycling particularly in aquatic ecosystems [19]. Proteobacteria have been identified as alpha-Gal positive [20] and are a major phylum of gram-negative bacteria that frequently function as microheterotrophs [21], thus suggesting the possibility that the alpha-Gal epitope was present in Proteobacteria within ancient stromatolites [22]. Other bacteria from Enterboacteriaceae (e.g. *Salmonella*, *Pseudomonas*, *Staphylococcus*), Rizobiaceae and Caulobacteriaceae families and within tick microbiome have been reported as alpha-Gal positive [22–24].

It is possible that alpha-Gal synthesis appeared and evolved in bacteria associated with stromatolites during the Mesoproterozoic Era ca. 1000 Mya (Figure 2). The Triassic period began after the Permian-Triassic extinction (known as “Great Dying”), which affected over 90% of Earth’s species. Life with new species then repopulated the planet with alpha-Gal-positive organisms, filling the empty ecological niches (https://www.nationalgeographic.com/science/article/triassic) with migration of multiples species including arthropods, birds, dinosaurs and plants during the Cretaceous period (Figure 2). Early insects with alpha-Gal synthesis belonging to modern groups of Diptera and Hymenoptera appeared in the Triassic led by recovery after the “Great Dying” extinction event. Acari of the group Eriophyoidea including the gall mites are also alpha-Gal positive and appeared during the Triassic. In the Eocene-Oligocene transition after end-Cretaceous extinction mainly caused by an asteroid impact, new species appeared and migrated to multiple regions worldwide.

At the end of Oligocene period (ca. 20 Mya), hominids evolved with a catastrophic selection event that resulted in the inactivation of the alpha-Gal synthesis, a capacity that appeared in Mammalia during Triassic (ca. 225 Mya) (Figure 2). This evolutionary adaptation in hominids was associated with the capacity to produce anti-alpha-Gal antibodies in response to gut microbiota, pathogens or ectoparasites with this modification with protective capacity against infectious diseases while sensitizing to the adverse effects of the AGS [12].

Improved fitness associated with alpha-Gal is theorized to be due to modulation of host immunity, anti-inflammatory effects, molecular mimicry of bioactive proteins and pathogen-host-vector interactions and disease transmission, functions particularly relevant in ectoparasites such as Ixodida ticks [12]. Additionally, other alpha-Gal positive Arthropoda such as Hymenoptera, Acari and Diptera together with Nematoda are also ectoparasites or predatory on mammals and may benefit from producing this biomolecule.

## Conclusions

In conclusion, evidence suggests the pre-Cambrian origin of alpha-Gal and supports the activation of alpha-Gal synthesis as an evolutionary adaptation in Mammalia and Arthropoda during the Triassic that improved fitness in multiple organisms but affected Hominidae leading to the catastrophic selection event in the Oligocene. This event inactivated 1,3-galactosyltransferase, GGTA1, and allowed hominids to develop anti-alpha-Gal antibodies with protective capacity against arthropods and pathogens with this modification but with risks of AGS. Additionally, environmental bacteria such as *Kluyvera* sp. and Alphaproteobacteria with this modification co-evolved with host species [25,26]. In the context of global rapidly changing climate and population dynamics, it is important to consider that multiple bacteria and arthropod species among other organisms evolved with the capacity to synthesize alpha-Gal. The synthesis of alpha-Gal in multiple species may increase the exposure to this biomolecule in humans and thus should be considered to reduce the risks of exposure to AGS. Future directions should focus on the study of the role of other organisms in addition to ticks in the evaluation and prevention of risks associated with the AGS (Table 1). As recently addressed [27,34], education for patients and medical staff is important to improve prevention and management of patients with AGS.

**Table 1.** Analysis of taxa with alpha-Gal negative and positive arthropoda and/or associated with the AGS.

Taxa	alpha-Gal negative	alpha-Gal positive	References
Scorpions Orthoptera Coleoptera	All negative	None known	[12]
Diptera	*Aedes* sp.	*Anopheles* sp. (*A. gambiae*) *Aedes aegypti*	[7,12,13]
	Muscidae	*Glossina* sp. (tsetse fly)	[8]
Acari	*Dermanyssus gallinae* (poultry red mite)	None known	Figure 1(A)
		Trombiculidae (chiggers)	[14,28]
Hymenoptera	*Vespa crabro* (European hornet) *Tetramorium* sp. (pavement ant)	*Vespula vulgaris* (common wasp) *Messor* sp. (harvest ant)	Figure 1(A)
Nematoda	*Toxocara canis*	*Parelaphostrongylus tenuis*	[11,12,29,30]
	*Ascaris suum*	*Haemonchus contortus*	
		*Ascaris lumbricoides*	[28,31,32]
		*Anisakis* sp.	[33]
Isoptera	Unknown	Unknown	
Ixodida	None known	Ticks (Ixodidae, Argasidae)	[15]

## Data Availability

All relevant data are within the manuscript. Further inquiries can be directed to the corresponding author.
